# Meteorological Register

**Published:** 1814-12

**Authors:** C. Blunt

**Affiliations:** Philosophical Instrument Maker, No. 38, Tavistock-Street, Covent-Garden


### METEOROLOGICAL REGISTER.

*From October the 25th, to November the 25th,* 1814.

Kept by C. BLUNT, Philosophical Instrument Maker, No. 38, Tavistock-Street, Covent-Garden.

| Moon. | Day. | Wind. | Barometrical Pressure. Max. | Min. | Mean. | Temperature. Max. | Min | Mean. | |
|---|---|---|---|---|---|---|---|---|---|
| | 26 | NW | 29·65 | 29 51 | 29·61 | 51 | 37 | 43·3 | Rain |
| | 27 | NW | 29 80 | 29·75 | 29·775 | 50 | 37 | 42·6 | Fair |
| | 28 | NW | 29·86 | 29·83 | 29·84 | 51 | 36 | 43 | Rain |
| ○ | 29 | SW | 29·78 | 29 67 | 29·727 | 52 | 37 | 44 | Cloudy |
| | 30 | SE | 29·86 | 29·84 | 29 85 | 51 | 34 | 43 | Fair |
| | 31 | SW | 29·90 | 29·89 | 29 892 | 52 | 43 | 48·6 | Foggy |
| | 1 | ESE | 29·94 | 29·93 | 29·932 | 50 | 42 | 46 | Cloudy |
| | 2 | NE | 29 97 | 29·96 | 29·965 | 46 | 38 | 43 | Cloudy |
| | 3 | NE | 29·92 | 29·88 | 29·895 | 47 | 35 | 41·3 | Cloudy |
| | 4 | E | 29·84 | 29 84 | 29 84 | 48 | 35 | 39·6 | Cloudy |
| ◐ | 5 | E | 29·78 | 29 74 | 29·75 | 47 | 37 | 39 6 | Cloudy |
| | 6 | NE | 29·74 | 29·74 | 29·74 | 48 | 36 | 39·6 | Fair |
| | 7 | N | 29·74 | 29·50 | 29·605 | 50 | 33 | 40 | Fair |
| | 8 | NW | 29·38 | 29·31 | 29·342 | 47 | 32 | 38·3 | Squall of rain, hail, & thund. at 4 P.M. |
| | 9 | W by N | 29·53 | 29·49 | 29·506 | 48 | 31 | 37·6 | Cloudy, snow, & hail, P.M. |
| | 10 | NW | 30·12 | 30·— | 30·056 | 44 | 30 | 35·6 | Frost. Fair |
| | 11 | W | 30·24 | 30·11 | 30·162 | 47 | 33 | 39 | Rain |
| | 12 | NW | 29 90 | 29 79 | 29 863 | 48 | 34 | 44 | Rain |
| | 13 | N | 29·84 | 29·84 | 29 84 | 48 | 37 | 44·6 | Cloudy |
| ● | 14 | W | 29 83 | 29 75 | 29 79 | 52 | 38 | 46 | Rain |
| | 15 | W | 29·85 | 29 60 | 29·746 | 55 | 45 | 50 | Rain |
| | 16 | W | 29 71 | 29·34 | 29·586 | 57 | 36 | 46 3 | Fair |
| | 17 | NW | 30·— | 29 99 | 29·995 | 56 | 42 | 47·6 | Stormy Wind Fair |
| | 18 | W | 29·75 | 29·64 | 29 713 | 56 | 44 | 51 | Rain |
| | 19 | NW | 29·57 | 29·44 | 29·775 | 49 | 33 | 44·6 | Fair |
| | 20 | NW | 29·5 | 29·5 | 29·5 | 45 | 35 | 41 | Rain |
| | 21 | NW | 29·65 | 29 6 | 29·87 | 45 | 25 | 36·3 | Clear |
| | 22 | NW | 29·69 | 29 69 | 29·69 | 35 | 25 | 29·6 | Clear |
| | 23 | NW | 29·70 | 29·66 | 29·67 | 44 | 26 | 33·6 | Fair. Fog. |
| | 24 | W | 29·80 | 29·70 | 29 742 | 44 | 32 | 38·6 | Foggy |
| | 25 | W | 29·64 | 29·42 | 29 77 | 53 | 40 | 46 6 | Rain |

RESULTS.

| Mean barometrical pressure of the month | | 29·654 | Mean temperature of the month | | 42·38 deg. |
|---|---|---|---|---|---|
| Maximum 30·24 | wind at | W | Maximum 57, | wind at | W |
| Minimum 29·31 | ——— | NW | Minimum 25, | ——— | NW |

*Scale exhibiting the prevailing Winds during the Month.*

| N | NE | E | SE | S | SW | W | NW |
|---|---|---|---|---|---|---|---|
| 2 | 3 | 2 | 2 | 0 | 2 | 7 | 13 |

| | Mean barometrical pressure. | Mean temperature. |
|---|---|---|
| From the full moon on the 28th Oct. to the last quarter on the 7th Nov. | 29·843 | 50·77 |
| ——— last quarter on the 7th, to the new moon on the 12th | 29·734 | 38·1 |
| ——— new moon on the 12th, to the first quarter on the 20th | 29·788 | 46·76 |

MONTHLY

